# 2-(1*H*-1,3-Benzodiazol-2-ylsulfan­yl)-1-(4-chloro­phen­yl)ethanone

**DOI:** 10.1107/S160053681103666X

**Published:** 2011-09-14

**Authors:** Hatem A. Abdel-Aziz, Seik Weng Ng, Edward R. T. Tiekink

**Affiliations:** a Department of Pharmaceutical Chemistry, College of Pharmacy, King Saud University, Riyadh 11451, Saudi Arabia; b Department of Chemistry, University of Malaya, 50603 Kuala Lumpur, Malaysia; c Chemistry Department, Faculty of, Science, King Abdulaziz University, PO Box 80203 Jeddah, Saudi Arabia

## Abstract

The mol­ecule in the structure of the title compound, C_15_H_11_ClN_2_OS, displays two planar residues [r.m.s. deviation = 0.014 Å for the benzimidazole residue, and the ketone group is co-planar with the benzene ring to which it is attached forming a O—C—C—C torsion angle of −173.18 (14) °] linked at the S atom. The overall shape is based on a twisted V, the dihedral angle formed between the two planes being 82.4 (2) °. The amine-H atom is bifurcated, forming N—H⋯O and N—H⋯S hydrogen bonds leading to dimeric aggregates. These are linked into a supra­molecular chain along the *c* axis *via* C—H⋯π hydrogen bonds. Chains form layers in the *ab* plane being connected along the *c* axis *via* weak π–π inter­actions [3.9578 (8) Å] formed between centrosymmetrically related chloro-substituted benzene rings.

## Related literature

For the biological and pharmacological properties of benzim­idazoles, see: Al-Rashood & Abdel-Aziz (2010 ▶); Abdel-Aziz *et al.* (2010 ▶). For the synthesis, see: Sarhan *et al.* (1996 ▶). For a related structure, see: Lynch & McClenaghan (2004 ▶).
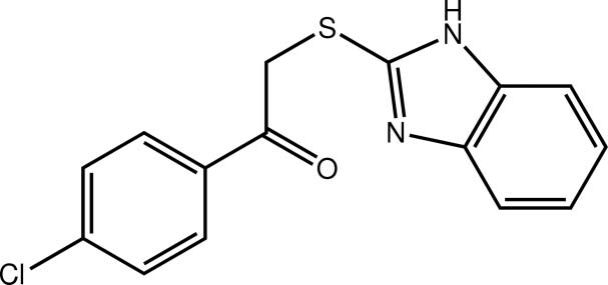


## Experimental

### 

#### Crystal data

C_15_H_11_ClN_2_OS
*M* *_r_* = 302.77
Monoclinic, 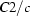

*a* = 27.3765 (4) Å
*b* = 9.2784 (2) Å
*c* = 10.3630 (2) Å
β = 93.087 (1)°
*V* = 2628.49 (9) Å^3^
*Z* = 8
Cu *K*α radiation
μ = 4.02 mm^−1^
*T* = 100 K
0.40 × 0.30 × 0.20 mm

#### Data collection

Agilent SuperNova Dual diffractometer with Atlas detector
Absorption correction: multi-scan (*CrysAlis PRO*; Agilent, 2010 ▶) *T*_min_ = 0.710, *T*_max_ = 1.000
5171 measured reflections
2613 independent reflections
2489 reflections with *I* > 2σ(*I*)
*R*_int_ = 0.016

#### Refinement

*R*[*F* ^2^ > 2σ(*F* ^2^)] = 0.029
*wR*(*F* ^2^) = 0.079
*S* = 1.07
2613 reflections
185 parameters
H atoms treated by a mixture of independent and constrained refinement
Δρ_max_ = 0.30 e Å^−3^
Δρ_min_ = −0.40 e Å^−3^

### 

Data collection: *CrysAlis PRO* (Agilent, 2010 ▶); cell refinement: *CrysAlis PRO*; data reduction: *CrysAlis PRO*; program(s) used to solve structure: *SHELXS97* (Sheldrick, 2008 ▶); program(s) used to refine structure: *SHELXL97* (Sheldrick, 2008 ▶); molecular graphics: *ORTEP-3* (Farrugia, 1997 ▶) and *DIAMOND* (Brandenburg, 2006 ▶); software used to prepare material for publication: *publCIF* (Westrip, 2010 ▶).

## Supplementary Material

Crystal structure: contains datablock(s) global, I. DOI: 10.1107/S160053681103666X/ez2258sup1.cif

Structure factors: contains datablock(s) I. DOI: 10.1107/S160053681103666X/ez2258Isup2.hkl

Supplementary material file. DOI: 10.1107/S160053681103666X/ez2258Isup3.cml

Additional supplementary materials:  crystallographic information; 3D view; checkCIF report

## Figures and Tables

**Table 1:** Hydrogen-bond geometry (Å, °) *Cg*1 and *Cg*2 are the centroids of the C10–C15 and N1,N2,C1,C6,C7 rings, respectively.

*D*—H⋯*A*	*D*—H	H⋯*A*	*D*⋯*A*	*D*—H⋯*A*
N2—H2⋯O1^i^	0.88 (2)	2.14 (2)	2.9104 (16)	144.9 (19)
N2—H2⋯S1^i^	0.88 (2)	2.69 (2)	3.4073 (12)	139.1 (16)
C8—H8a⋯*Cg*1^ii^	0.99	2.89	3.5678 (15)	126
C8—H8b⋯*Cg*2^iii^	0.99	2.76	3.4204 (16)	125

Symmetry codes: (i) 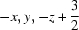; (ii) 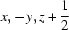; (iii) 
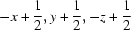
.

**Fig. 1.**
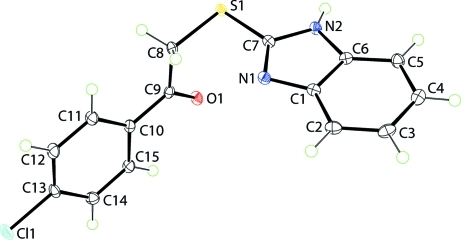
The molecular structure of (I) showing the atom-labelling scheme and displacement ellipsoids at the 50% probability level.

**Fig. 2.**
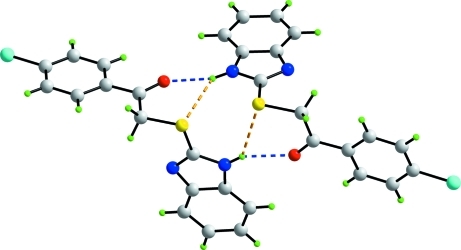
Dimeric aggregates with 2-fold symmetry and sustained by N—H···O and N—H···S hydrogen bonds, shown as blue and orange dashed lines, respectively.

**Fig. 3.**
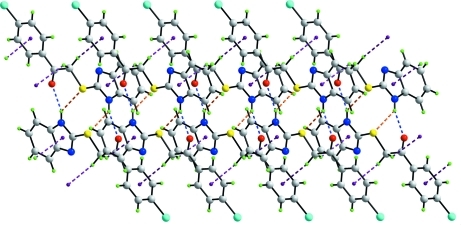
Supramolecular chain in (I) whereby the dimeric aggregates shown in Fig. 2 are connected by C—H···π interactions (purple dashed lines), The N—H···O and N—H···S hydrogen bonds are shown as blue and orange dashed lines, respectively.

**Fig. 4.**
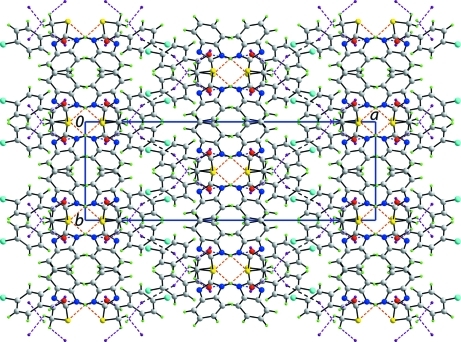
A view in projection down the c axis of the unit-cell contents of (I). The N—H···O, N—H···S and C—H···π interactions are shown as blue, orange and purple dashed lines, respectively.

## supplementary crystallographic information

### Comment

The structural analysis of the title compound, (I), is motivated by recent studies into the biological potential of benzimidazoles (Al-Rashood & Abdel-Aziz, 2010; Abdel-Aziz *et al.*, 2010). The molecule of (I), Fig. 1, has a twisted V-shape. As expected, the benzimidazole residue is planar (r.m.s. deviation = 0.014 Å). The ketone group is co-planar with the benzene ring to which it is attached as seen in the value of the O1—C9—C10—C11 torsion angle of -173.18 (14) °. As the S1—C8—C9—O1 torsion angle is -0.39 (18) °, the molecule comprises two planar residues that form a dihedral angle of 82.4 (2) °. The most closely related structure in the literature is that of 2-(benzoylmethylsulfanyl)-6-methoxy-1*H*-benzimidazole (Lynch & McClenaghan, 2004), *i.e*. with a methoxy substituent on the benzene ring of the benzimidazole and no substituent on the ring attached to the ketone. This adopts a similar conformation with the ketone benzene ring inclined to the benzimidazole residue with the dihedral angle formed between the ring systems being 67.13 (9) °.

In the crystal packing two molecules, related by a 2-fold axis of symmetry associate *via* N—H···O and N—H···S hydrogen bonds as the amine-H atom is bifurcated, Table 1. As seen from Fig. 2, this results in the formation of two *S*(5), {···H···OC_2_S} ring motifs which flank a central eight-membered {···HNCS}_2_ synthon. The dimeric aggregates are linked into a supramolecular chain along the *c* axis *via* C—H···π interactions Table 1 and Fig. 3. Chains assemble into layers in the *ab* plane and are connected along the *c* axis *via* weak π···π interactions of 3.9578 (8) Å formed between the chloro-substituted benzene rings (C10–C15); symmetry operation: 1/2 - *x*, 1/2 - *y*, 1 - *z*, Fig. 4.

### Experimental

The reaction of 2-mercaptobenzimidazole with 4-chloroacetophenone in boiling AcOH/H_2_SO_4_ afforded the sulfate salt of 2-(1*H*-benzo[*d*]imidazol-2-ylthio)-1-(4-chlorophenyl)ethanone after Sarhan *et al.* (1996). Neutralization of the latter salt afforded the title compound and the light-brown crystals were grown from its ethanol solution by slow evaporation at room temperature.

### Refinement

Carbon-bound H-atoms were placed in calculated positions [C—H 0.95 to 0.99 Å, *U*_iso_(H) = 1.2*U*_eq_(C)] and were included in the refinement in the riding model approximation. The amino-H atom was located in a difference Fourier map, and subsequently refined freely.

### Crystal data

**Table:**

C_15_H_11_ClN_2_OS	*F*(000) = 1248
*M**_r_* = 302.77	*D*_x_ = 1.530 Mg m^−^^3^
Monoclinic, *C*2/*c*	Cu *K*α radiation, λ = 1.5418 Å
Hall symbol: -C 2yc	Cell parameters from 3792 reflections
*a* = 27.3765 (4) Å	θ = 3.2–74.1°
*b* = 9.2784 (2) Å	µ = 4.02 mm^−^^1^
*c* = 10.3630 (2) Å	*T* = 100 K
β = 93.087 (1)°	Block, light-brown
*V* = 2628.49 (9) Å^3^	0.40 × 0.30 × 0.20 mm
*Z* = 8	

### Data collection

**Table:**

Agilent SuperNova Dual diffractometer with Atlas detector	2613 independent reflections
Radiation source: SuperNova (Cu) X-ray Source	2489 reflections with *I* > 2σ(*I*)
mirror	*R*_int_ = 0.016
Detector resolution: 10.4041 pixels mm^-1^	θ_max_ = 74.2°, θ_min_ = 3.2°
ω scan	*h* = −32→34
Absorption correction: multi-scan (*CrysAlis PRO*; Agilent, 2010)	*k* = −11→10
*T*_min_ = 0.710, *T*_max_ = 1.000	*l* = −9→12
5171 measured reflections	

### Refinement

**Table:**

Refinement on *F*^2^	Primary atom site location: structure-invariant direct methods
Least-squares matrix: full	Secondary atom site location: difference Fourier map
*R*[*F*^2^ > 2σ(*F*^2^)] = 0.029	Hydrogen site location: inferred from neighbouring sites
*wR*(*F*^2^) = 0.079	H atoms treated by a mixture of independent and constrained refinement
*S* = 1.07	*w* = 1/[σ^2^(*F*_o_^2^) + (0.0442*P*)^2^ + 2.8814*P*] where *P* = (*F*_o_^2^ + 2*F*_c_^2^)/3
2613 reflections	(Δ/σ)_max_ = 0.001
185 parameters	Δρ_max_ = 0.30 e Å^−^^3^
0 restraints	Δρ_min_ = −0.40 e Å^−^^3^

### Special details

**Table:**

Geometry. All e.s.d.'s (except the e.s.d. in the dihedral angle between two l.s. planes) are estimated using the full covariance matrix. The cell e.s.d.'s are taken into account individually in the estimation of e.s.d.'s in distances, angles and torsion angles; correlations between e.s.d.'s in cell parameters are only used when they are defined by crystal symmetry. An approximate (isotropic) treatment of cell e.s.d.'s is used for estimating e.s.d.'s involving l.s. planes.
Refinement. Refinement of *F*^2^ against ALL reflections. The weighted *R*-factor *wR* and goodness of fit *S* are based on *F*^2^, conventional *R*-factors *R* are based on *F*, with *F* set to zero for negative *F*^2^. The threshold expression of *F*^2^ > σ(*F*^2^) is used only for calculating *R*-factors(gt) *etc*. and is not relevant to the choice of reflections for refinement. *R*-factors based on *F*^2^ are statistically about twice as large as those based on *F*, and *R*- factors based on ALL data will be even larger.

### Fractional atomic coordinates and isotropic or equivalent isotropic displacement parameters (Å^2^)

**Table:**

	*x*	*y*	*z*	*U*_iso_*/*U*_eq_	
Cl1	0.282969 (13)	0.21419 (4)	0.22654 (4)	0.01886 (12)	
S1	0.059988 (12)	−0.01719 (4)	0.74205 (3)	0.01091 (11)	
O1	0.07341 (4)	0.18383 (12)	0.52818 (10)	0.0138 (2)	
N1	0.10732 (4)	0.20841 (14)	0.86384 (12)	0.0121 (3)	
N2	0.03011 (4)	0.17240 (14)	0.92176 (12)	0.0118 (3)	
H2	−0.0005 (8)	0.142 (2)	0.9163 (19)	0.026 (5)*	
C1	0.09605 (5)	0.30740 (16)	0.95916 (13)	0.0111 (3)	
C2	0.12521 (5)	0.41479 (17)	1.01856 (14)	0.0147 (3)	
H2A	0.1576	0.4315	0.9937	0.018*	
C3	0.10522 (6)	0.49602 (17)	1.11487 (15)	0.0156 (3)	
H3	0.1244	0.5692	1.1570	0.019*	
C4	0.05726 (6)	0.47245 (17)	1.15152 (15)	0.0159 (3)	
H4	0.0448	0.5299	1.2181	0.019*	
C5	0.02753 (5)	0.36737 (17)	1.09304 (14)	0.0141 (3)	
H5	−0.0050	0.3517	1.1173	0.017*	
C6	0.04807 (5)	0.28607 (15)	0.99671 (13)	0.0109 (3)	
C7	0.06706 (5)	0.13180 (16)	0.84568 (13)	0.0106 (3)	
C8	0.11504 (5)	0.00291 (16)	0.65706 (14)	0.0115 (3)	
H8A	0.1421	0.0295	0.7196	0.014*	
H8B	0.1233	−0.0909	0.6184	0.014*	
C9	0.11099 (5)	0.11594 (15)	0.55116 (13)	0.0109 (3)	
C10	0.15473 (5)	0.13996 (16)	0.47401 (13)	0.0109 (3)	
C11	0.19656 (5)	0.05349 (17)	0.48894 (14)	0.0142 (3)	
H11	0.1980	−0.0211	0.5518	0.017*	
C12	0.23593 (5)	0.07600 (17)	0.41252 (15)	0.0153 (3)	
H12	0.2642	0.0168	0.4222	0.018*	
C13	0.23353 (5)	0.18575 (17)	0.32202 (14)	0.0133 (3)	
C14	0.19256 (5)	0.27407 (16)	0.30626 (14)	0.0145 (3)	
H14	0.1916	0.3498	0.2445	0.017*	
C15	0.15315 (5)	0.25001 (17)	0.38202 (14)	0.0129 (3)	
H15	0.1248	0.3089	0.3713	0.015*	

### Atomic displacement parameters (Å^2^)

**Table:**

	*U*^11^	*U*^22^	*U*^33^	*U*^12^	*U*^13^	*U*^23^
Cl1	0.01085 (18)	0.0243 (2)	0.0221 (2)	0.00010 (14)	0.00726 (14)	0.00128 (14)
S1	0.00850 (18)	0.01265 (19)	0.01167 (18)	−0.00049 (12)	0.00121 (12)	−0.00085 (12)
O1	0.0078 (5)	0.0169 (5)	0.0168 (5)	0.0032 (4)	0.0010 (4)	0.0014 (4)
N1	0.0084 (6)	0.0141 (6)	0.0138 (6)	0.0000 (5)	0.0005 (4)	0.0004 (5)
N2	0.0071 (6)	0.0149 (6)	0.0134 (6)	−0.0017 (5)	0.0011 (4)	−0.0022 (5)
C1	0.0088 (6)	0.0125 (7)	0.0120 (6)	0.0005 (5)	−0.0011 (5)	0.0026 (5)
C2	0.0099 (6)	0.0154 (7)	0.0184 (7)	−0.0025 (6)	−0.0017 (5)	0.0021 (6)
C3	0.0151 (7)	0.0125 (7)	0.0185 (7)	−0.0023 (6)	−0.0054 (6)	0.0001 (6)
C4	0.0176 (7)	0.0147 (7)	0.0152 (7)	0.0019 (6)	−0.0008 (6)	−0.0019 (6)
C5	0.0113 (7)	0.0162 (7)	0.0150 (7)	0.0004 (6)	0.0018 (5)	−0.0008 (6)
C6	0.0096 (6)	0.0110 (7)	0.0118 (6)	−0.0004 (5)	−0.0019 (5)	0.0009 (5)
C7	0.0084 (6)	0.0131 (7)	0.0101 (6)	0.0009 (5)	−0.0004 (5)	0.0018 (5)
C8	0.0080 (6)	0.0146 (7)	0.0120 (6)	0.0016 (5)	0.0017 (5)	−0.0004 (5)
C9	0.0097 (7)	0.0111 (7)	0.0118 (6)	0.0001 (5)	−0.0006 (5)	−0.0038 (5)
C10	0.0076 (6)	0.0130 (7)	0.0122 (6)	−0.0005 (5)	−0.0007 (5)	−0.0032 (5)
C11	0.0112 (7)	0.0156 (7)	0.0158 (7)	0.0026 (6)	0.0007 (5)	0.0016 (6)
C12	0.0090 (7)	0.0173 (8)	0.0195 (7)	0.0037 (6)	0.0004 (5)	−0.0002 (6)
C13	0.0081 (6)	0.0176 (7)	0.0143 (7)	−0.0025 (6)	0.0025 (5)	−0.0037 (6)
C14	0.0123 (7)	0.0143 (7)	0.0168 (7)	−0.0006 (6)	0.0000 (6)	0.0013 (6)
C15	0.0086 (6)	0.0138 (7)	0.0162 (7)	0.0018 (6)	−0.0012 (5)	−0.0008 (6)

### Geometric parameters (Å, °)

**Table:**

Cl1—C13	1.7392 (15)	C5—C6	1.394 (2)
S1—C7	1.7548 (15)	C5—H5	0.9500
S1—C8	1.7958 (14)	C8—C9	1.518 (2)
O1—C9	1.2187 (17)	C8—H8A	0.9900
N1—C7	1.3165 (19)	C8—H8B	0.9900
N1—C1	1.3955 (19)	C9—C10	1.4920 (19)
N2—C7	1.3685 (18)	C10—C15	1.396 (2)
N2—C6	1.3843 (19)	C10—C11	1.400 (2)
N2—H2	0.88 (2)	C11—C12	1.387 (2)
C1—C2	1.399 (2)	C11—H11	0.9500
C1—C6	1.404 (2)	C12—C13	1.384 (2)
C2—C3	1.386 (2)	C12—H12	0.9500
C2—H2A	0.9500	C13—C14	1.392 (2)
C3—C4	1.403 (2)	C14—C15	1.386 (2)
C3—H3	0.9500	C14—H14	0.9500
C4—C5	1.388 (2)	C15—H15	0.9500
C4—H4	0.9500		
			
C7—S1—C8	98.65 (7)	C9—C8—H8A	108.9
C7—N1—C1	103.95 (12)	S1—C8—H8A	108.9
C7—N2—C6	106.36 (12)	C9—C8—H8B	108.9
C7—N2—H2	127.0 (14)	S1—C8—H8B	108.9
C6—N2—H2	126.0 (14)	H8A—C8—H8B	107.7
N1—C1—C2	129.61 (13)	O1—C9—C10	120.76 (13)
N1—C1—C6	110.45 (12)	O1—C9—C8	121.77 (13)
C2—C1—C6	119.92 (13)	C10—C9—C8	117.46 (12)
C3—C2—C1	117.78 (14)	C15—C10—C11	119.33 (13)
C3—C2—H2A	121.1	C15—C10—C9	118.61 (13)
C1—C2—H2A	121.1	C11—C10—C9	122.04 (13)
C2—C3—C4	121.42 (14)	C12—C11—C10	120.44 (14)
C2—C3—H3	119.3	C12—C11—H11	119.8
C4—C3—H3	119.3	C10—C11—H11	119.8
C5—C4—C3	121.76 (14)	C11—C12—C13	119.20 (14)
C5—C4—H4	119.1	C11—C12—H12	120.4
C3—C4—H4	119.1	C13—C12—H12	120.4
C4—C5—C6	116.30 (13)	C12—C13—C14	121.44 (14)
C4—C5—H5	121.8	C12—C13—Cl1	119.16 (11)
C6—C5—H5	121.8	C14—C13—Cl1	119.40 (12)
N2—C6—C5	132.02 (14)	C15—C14—C13	119.06 (14)
N2—C6—C1	105.14 (12)	C15—C14—H14	120.5
C5—C6—C1	122.81 (14)	C13—C14—H14	120.5
N1—C7—N2	114.09 (13)	C14—C15—C10	120.53 (13)
N1—C7—S1	125.23 (11)	C14—C15—H15	119.7
N2—C7—S1	120.58 (11)	C10—C15—H15	119.7
C9—C8—S1	113.32 (10)		
			
C7—N1—C1—C2	178.86 (15)	C8—S1—C7—N1	−11.63 (14)
C7—N1—C1—C6	0.28 (15)	C8—S1—C7—N2	172.27 (11)
N1—C1—C2—C3	−177.76 (14)	C7—S1—C8—C9	−79.85 (11)
C6—C1—C2—C3	0.7 (2)	S1—C8—C9—O1	−0.39 (18)
C1—C2—C3—C4	−0.4 (2)	S1—C8—C9—C10	−179.50 (10)
C2—C3—C4—C5	−0.2 (2)	O1—C9—C10—C15	5.2 (2)
C3—C4—C5—C6	0.4 (2)	C8—C9—C10—C15	−175.68 (13)
C7—N2—C6—C5	−178.12 (15)	O1—C9—C10—C11	−173.18 (14)
C7—N2—C6—C1	0.27 (15)	C8—C9—C10—C11	5.9 (2)
C4—C5—C6—N2	178.08 (15)	C15—C10—C11—C12	−0.5 (2)
C4—C5—C6—C1	−0.1 (2)	C9—C10—C11—C12	177.87 (13)
N1—C1—C6—N2	−0.35 (16)	C10—C11—C12—C13	0.5 (2)
C2—C1—C6—N2	−179.09 (13)	C11—C12—C13—C14	0.1 (2)
N1—C1—C6—C5	178.23 (13)	C11—C12—C13—Cl1	179.83 (12)
C2—C1—C6—C5	−0.5 (2)	C12—C13—C14—C15	−0.8 (2)
C1—N1—C7—N2	−0.10 (16)	Cl1—C13—C14—C15	179.50 (11)
C1—N1—C7—S1	−176.42 (10)	C13—C14—C15—C10	0.8 (2)
C6—N2—C7—N1	−0.12 (17)	C11—C10—C15—C14	−0.2 (2)
C6—N2—C7—S1	176.39 (10)	C9—C10—C15—C14	−178.60 (13)

### Hydrogen-bond geometry (Å, °)

**Table:**

Cg1 and Cg2 are the centroids of the C10–C15 and N1,N2,C1,C6,C7 rings, respectively.

**Table:**

*D*—H···*A*	*D*—H	H···*A*	*D*···*A*	*D*—H···*A*
N2—H2···O1^i^	0.88 (2)	2.14 (2)	2.9104 (16)	144.9 (19)
N2—H2···S1^i^	0.88 (2)	2.69 (2)	3.4073 (12)	139.1 (16)
C8—H8a···Cg1^ii^	0.99	2.89	3.5678 (15)	126
C8—H8b···Cg2^iii^	0.99	2.76	3.4204 (16)	125

Symmetry codes: (i) −*x*, *y*, −*z*+3/2; (ii) *x*, −*y*, *z*+1/2; (iii) −*x*+1/2, *y*+1/2, −*z*+1/2.

## References

[bb1] Abdel-Aziz, H. A., Saleh, T. S. & El-Zahabi, H. S. A. (2010). *Arch. Pharm.* **343**, 24–30. 10.1002/ardp.200900082 19921685

[bb2] Agilent (2010). *CrysAlis PRO* Agilent Technologies, Yarnton, England.

[bb3] Al-Rashood, K. A. & Abdel-Aziz, H. A. (2010). *Molecules*, **15**, 3775–3815. 10.3390/molecules15063775 PMC6264459 20657409

[bb4] Brandenburg, K. (2006). *DIAMOND* Crystal Impact GbR, Bonn, Germany.

[bb5] Farrugia, L. J. (1997). *J. Appl. Cryst.* **30**, 565.

[bb6] Lynch, D. E. & McClenaghan, I. (2004). *Acta Cryst.* E**60**, o363–o364.

[bb7] Sarhan, A. A. O., El-Shereif, H. A. H. & Mahmoud, A. M. (1996). *Tetrahedron*, **52**, 10485–10496.

[bb8] Sheldrick, G. M. (2008). *Acta Cryst.* A**64**, 112–122. 10.1107/S0108767307043930 18156677

[bb9] Westrip, S. P. (2010). *J. Appl. Cryst.* **43**, 920–925.

